# Mapping emotional patterns in linear recreational landscapes: Spatial structure, positive-negative co-occurrence patterns, and nonlinear drivers

**DOI:** 10.1371/journal.pone.0356187

**Published:** 2026-09-08

**Authors:** Minlong Yan, Chengchao Yu, Qinlan Lin, Zhichao Huang, Yanru Duan, Liying Zhu

**Affiliations:** 1 Landscape Architecture and Art College, Fujian Agriculture and Forestry University, Fuzhou, Fujian, China; 2 State Key Laboratory of Resources and Environmental Information System, Institute of Geographic Sciences and Natural Resources Research, Chinese Academy of Sciences, Beijing, China; Zhejiang A and F University, CHINA

## Abstract

As landscape planning shifts toward experience-oriented design, emotion mapping has become critical to understanding how spatial environments shape human perception. However existing approaches are largely grounded in site-based or area-based spatial units, limiting their ability to represent emotional variation in directional and continuous linear recreational landscapes. To address this gap, this study develops an integrated framework combining panoramic visual capture, VR-based emotion elicitation experiments, and MaxEnt spatial prediction modeling to quantify and map emotional fluctuations along linear landscapes. Using the No. 1 Scenic Road of Wuyi Mountain National Park as a case study, we find that: (1) emotional fluctuations exhibit a hierarchical spatial structure, clustering along the main road and gradually diffusing into surrounding buffer zones; (2) positive and negative emotions significantly co-occur in core scenic segments, challenging the traditional assumption of emotional mutual exclusivity; (3) emotional distributions are jointly influenced by multiple landscape attributes, with key drivers demonstrating pronounced nonlinear threshold effects. This study advances emotional mapping from static area-based settings to continuous linear recreational systems and provides evidence-based support for refined landscape planning and management.

## 1. Introduction

In recent decades, landscape planning has gradually shifted from function-oriented provision to experience-oriented design [1]. Beyond ecological performance and spatial efficiency, landscapes are increasingly expected to foster meaningful perceptual and emotional experiences [2]. The importance of experiencing and understanding the management of natural systems through the emotional and perceptual relationships people develop with nature was emphasized by scholars in early landscape research [3,4]. This idea was further developed in subsequent studies, highlighting the key role of emotional experience in environmental management [5]. Emotional responses influence aesthetic evaluation, recreational satisfaction, and place attachment, and thus play a pivotal role in shaping human-environment interactions. Understanding how spatial environments structure emotional experience has therefore become a critical concern in contemporary landscape research and practice.

To spatially represent emotional experience, emotion mapping has emerged as a widely adopted analytical framework [6]. Previous studies have successfully identified emotional hotspots and restorative environments in parks, neighborhoods, and urban districts [7–9]. As a tool for landscape planning evaluation and participatory planning, emotion mapping provides decision support for planners and integrates the emotional dimensions of users into spatial optimization considerations [9]. Methodologically, approaches range from participatory annotation and questionnaire-based surveys [10,11] to social media sentiment analysis [12,13], and precise measurements in controlled laboratory settings [14]. However, despite these advances, most emotion mapping research remains grounded in site-based or area-based spatial assumptions, where emotional observations are treated as discrete and relatively static phenomena.

Scenic roads represent a typical form of linear recreational landscape [15,16]. Unlike traditional recreational spaces such as parks and reserves, they are characterized by continuous, directional, and movement-dependent experiences, in which landscape perception evolves progressively along the travel route rather than within a fixed location [2,17–19]. Previous studies in environmental perception have shown that landscape experience is shaped not only by the characteristics of individual scenes but also by the sequential organization of views encountered during movement. Accordingly, environmental stimuli change progressively along the route, and emotional responses fluctuate within a path-dependent spatial continuum [4,20–22]. Therefore, analytical frameworks developed for relatively enclosed park environments, which typically assume static observation conditions, are less suitable for capturing the dynamic emotional variations associated with continuous scenic-road experiences [23].

When applied to linear recreational spaces, conventional data collection and modeling approaches encounter significant limitations. On-site surveys become spatially fragmented and resource-intensive over long corridors [24], while social media data often suffer from sampling bias and discontinuous spatial coverage [25]. More fundamentally, existing frameworks lack a systematic way to transform discrete emotional observations into continuous probability surfaces suitable for linear spatial systems. As a result, spatial prediction of emotions in linear recreational landscapes remains under explored. Beyond these methodological gaps, existing research often assumes that positive and negative emotions are spatially separated [9]. They rarely provide systematic discussions about the spatial coexistence of positive and negative emotions. Furthermore, the relationship between emotions and landscape elements is frequently treated as linear, overlooking nonlinear responses and threshold effects [26]. Consequently, these limitations hinder a deeper understanding of emotional mechanisms in linear recreational spaces, making it difficult to translate research findings into experience-based planning practices.

To address these gaps, this study takes the No. 1 Scenic Road of Wuyi Mountain National Park as a representative linear recreational landscape. We develop an integrated framework that combines panoramic visual capture, VR-based emotion experiments, and MaxEnt spatial prediction models to convert discrete emotional responses into continuous linear probability surfaces. By adapting emotion mapping to a path-dependent spatial system, this study extends the methodological scope of emotional mapping from static area-based contexts to directional linear landscapes. The findings provide methodological support for experience-oriented landscape planning and contribute to a deeper understanding of emotional organization in linear recreational systems.

## 2. Materials and methods

### 2.1. Research frameworks

**Fig 1:**
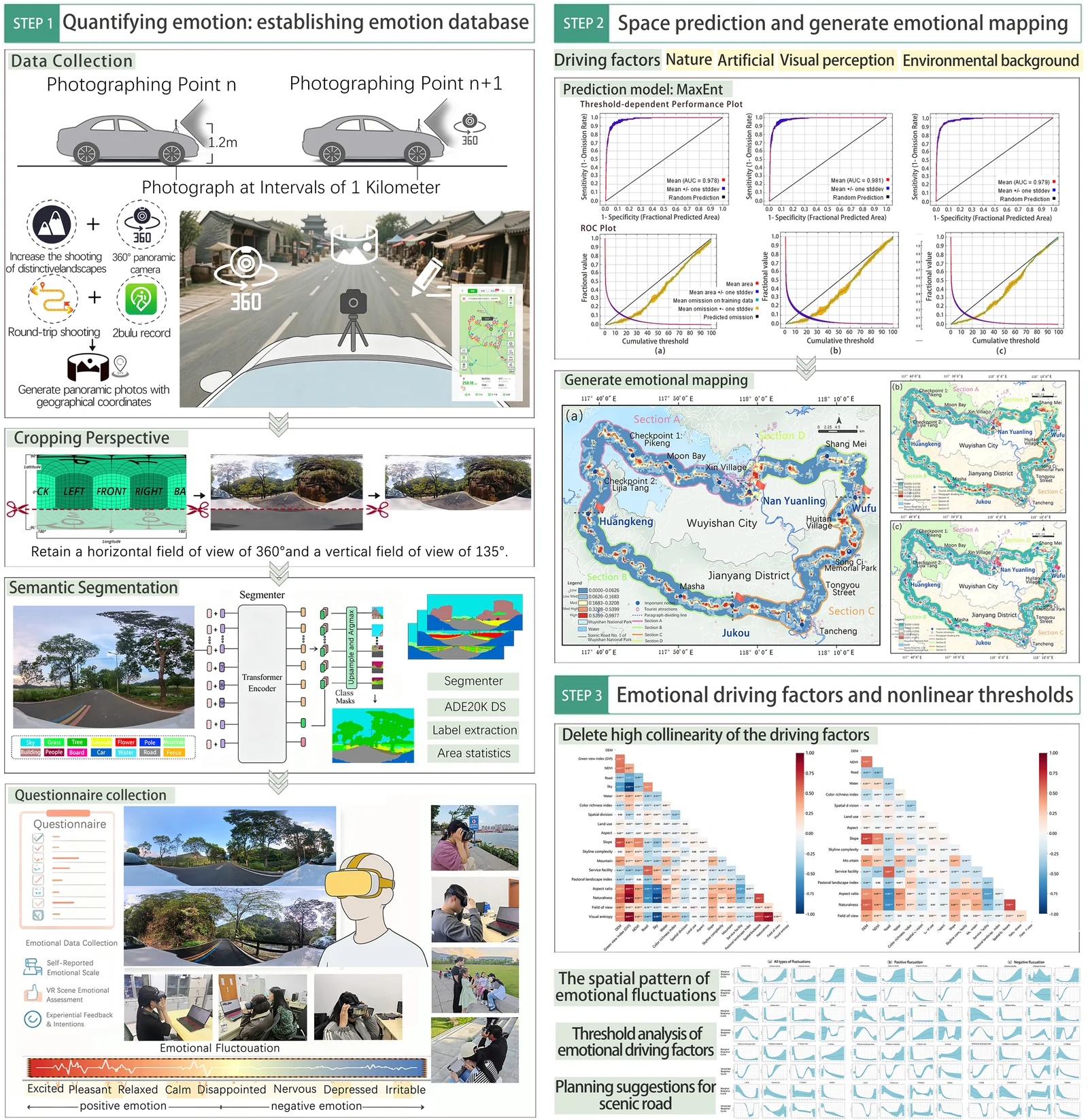
Research frameworks.

The research framework consists of three main stages (Fig 1).

First, emotional quantification. Panoramic images of the scenic road were collected and processed, and semantic segmentation was performed on the data from the scenic road using the ADE20K dataset to obtain the basic emotional data. Next, an emotional database was established through a questionnaire-based virtual reality (VR) experiment. Participants wore VR headsets to observe the captured scenes while their psychological responses were recorded to capture emotional fluctuations.

Second, spatial prediction. The MaxEnt prediction model was employed to predict emotional fluctuations within the scenic road and its 2-km buffer zone, and then generate the final emotional mapping.

Finally, driving mechanism analysis. Based on the results of the emotional mapping, the study explored the emotional driving factors and nonlinear threshold mechanisms, proposing targeted planning strategies and management interventions.

### 2.2. Study area overview

**Fig 2:**
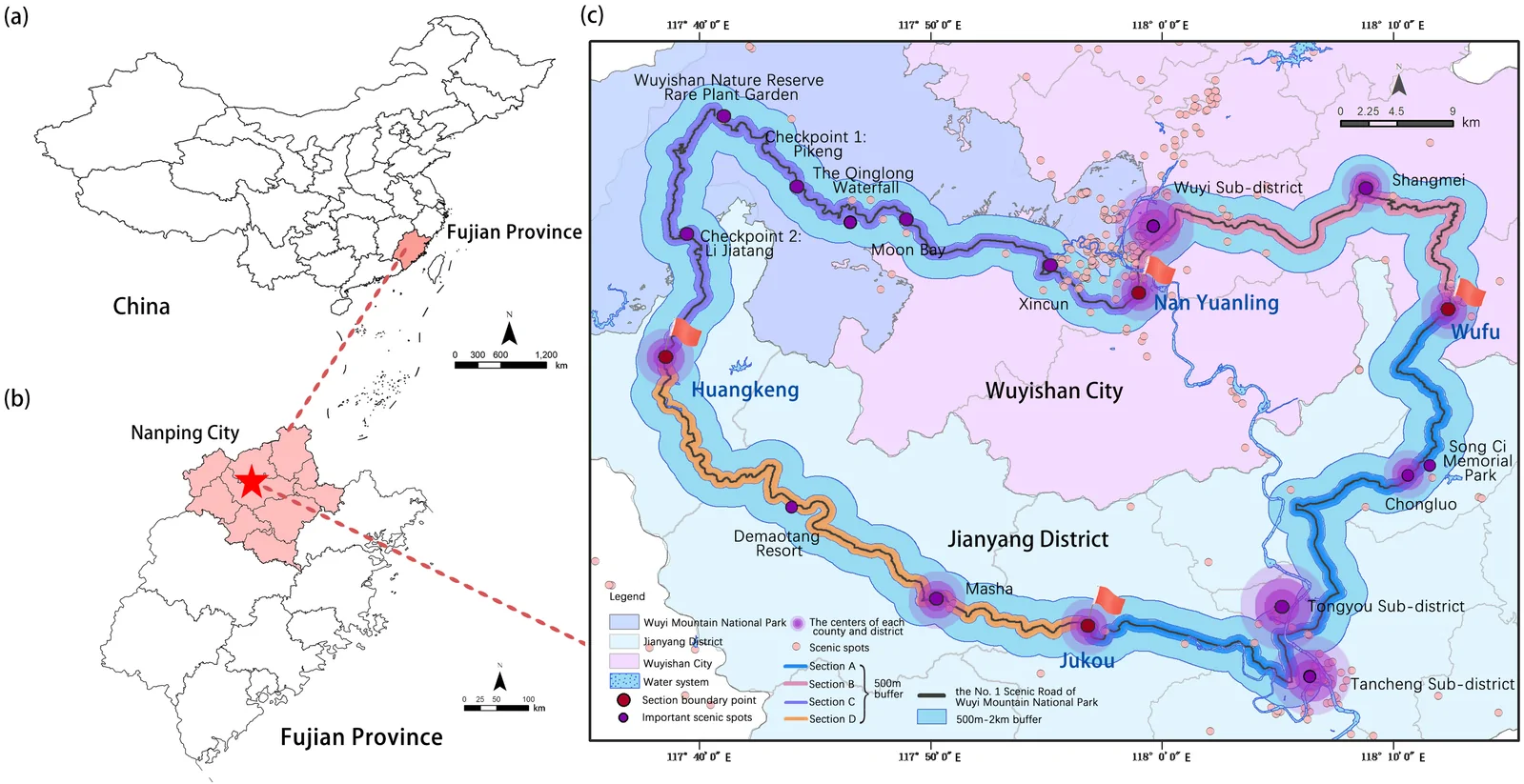
Overview of the study area. (a) China. (b) Nanping City, Fujian Province. (c) The No. 1 Scenic Road of Wuyi Mountain National Park.

The No. 1 Scenic Road of Wuyi Mountain National Park (hereinafter referred to as “the No. 1 Scenic Road”) is the first national park scenic road in China (Fig 2). Located in the northwestern part of Fujian Province, it traverses Wuyi Mountain National Park and its adjacent collaborative zones. With a total length of approximately 257 km, this loop-shaped route encompasses 11 towns and villages within Wuyishan City and Jianyang District. The road serves as a linear eco-cultural corridor designed to integrate ecological conservation, cultural heritage display, and recreational experiences, effectively linking the park’ s core protection zones, general control zones, and surrounding traditional villages and eco-agricultural areas. Along the road, nearly 60 key landscape and recreational nodes are distributed, showcasing multiple natural and cultural landscape features of Wuyi Mountain, such as Danxia landforms, the Nine Bend Stream, traditional villages, and tea culture. The road forms a complete vertical sequence, transitioning from a high-altitude forest ecosystem to a low-altitude valley agricultural landscape [27].

The No. 1 Scenic Road was selected as the study area because it exhibits the key characteristics of a typical linear recreational landscape, including continuous route-based travel, sequential landscape perception, and diverse natural landscape types within a single corridor. These characteristics make it an appropriate case for investigating the spatial dynamics of visitors’ emotional responses during continuous movement, while also providing methodological insights that may be transferable to other scenic-road environments with similar spatial characteristics. As an important spatial link between ecological protection and sustainable tourism, the No. 1 Scenic Road provides a representative case for investigating emotional experiences and spatial response mechanisms in linear recreational spaces within the national park system. Based on differences in terrain conditions and landscape features, the entire route is divided into four sections: Section A (Nan Yuanling to Huangkeng Town), Section B (Huangkeng Town to Jukou Town), Section C (Jukou Town to Wufu Town), Section D (Wufu Town to Nan Yuanling).

### 2.3. Equidistant bidirectional collection method

In the field of scenic road landscape quality assessment, with reference to relevant studies, this study adopted a 1 km sampling interval considering the need to characterize the continuity of the road landscape and minimize the repetition of landscape features [17,28,29].

**Fig 3:**
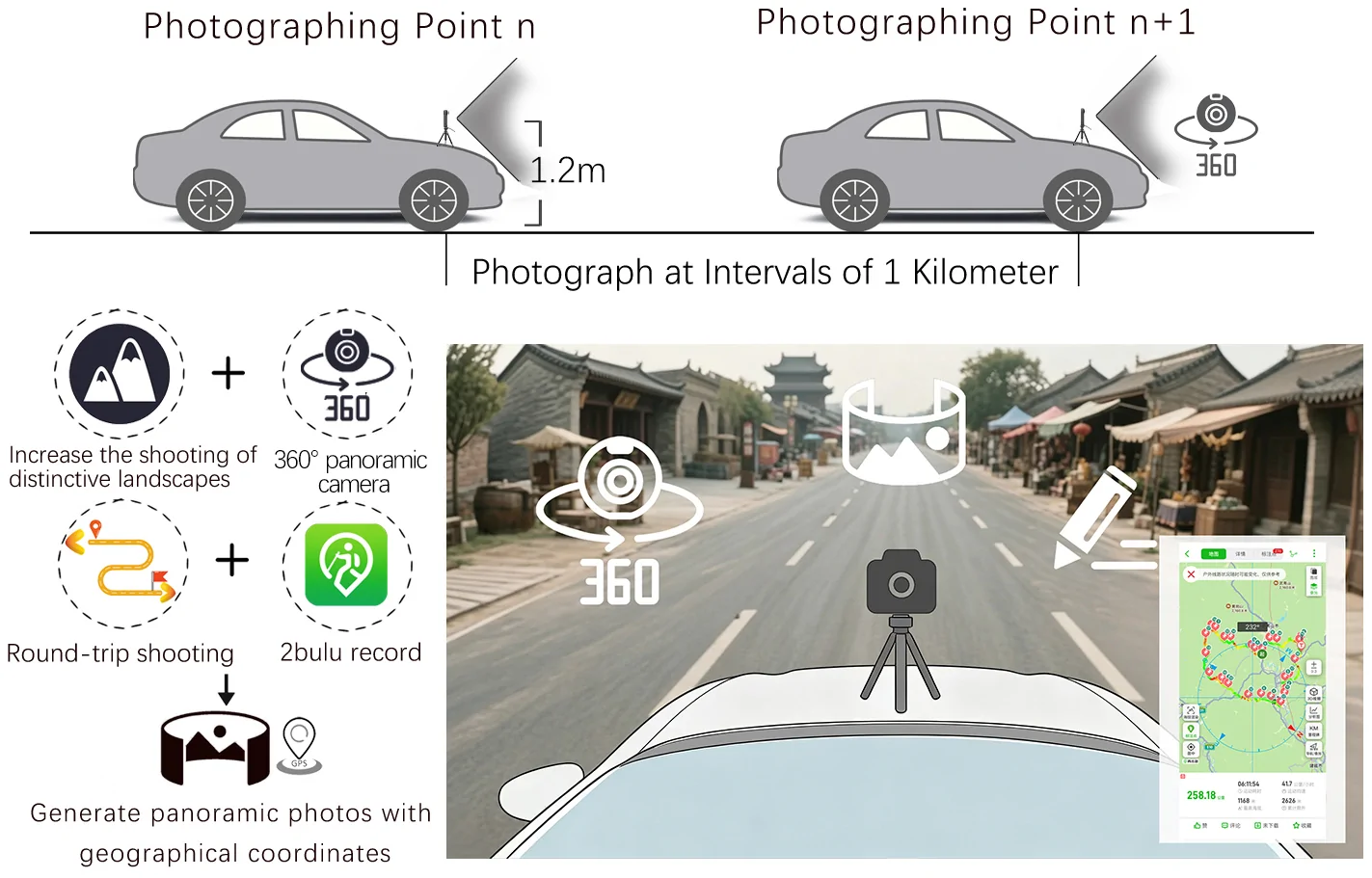
Showcase of the No.1 Scenic Road data collection method.

Taking the No. 1 Scenic Road as the empirical case, the study defined the segment between the entry and exit points at Nanyuanling Station as the specific research scope. Field data collection was conducted on public roads within the Wuyi Mountain National Park and involved only non-invasive, non-destructive observation and photography of the landscape. To simulate authentic driving visual perceptions, an Insta360 X3 panoramic camera was mounted on the vehicle hood at a height of 1.2 m above the ground [17]. Following the 1 km equidistant sampling principle, supplementary shots were taken at significant cultural and natural landmarks.The camera’ s GPS function, integrated with the “2bulu” trajectory tracking software, was used to record precise geographic coordinates and driving data for each panoramic image (Fig 3). A total of 624 raw panoramic photographs were collected. Following a quality screening process to exclude blurred or poorly illuminated samples, a final dataset of 614 high-resolution panoramic images was retained, consisting of 336 outward and 278 return trip images. This dataset addresses the lack of street-view data in the study area. Subsequently, these images were standardized to a FOV of 360° horizontally and 135° vertically. These parameters align with the human binocular FOV (approximately 180° horizontal and 125° vertical), enabling a more comprehensive and vivid restoration of the authentic spatial scenes along the scenic road.

### 2.4. Semantic segmentation

**Fig 4:**
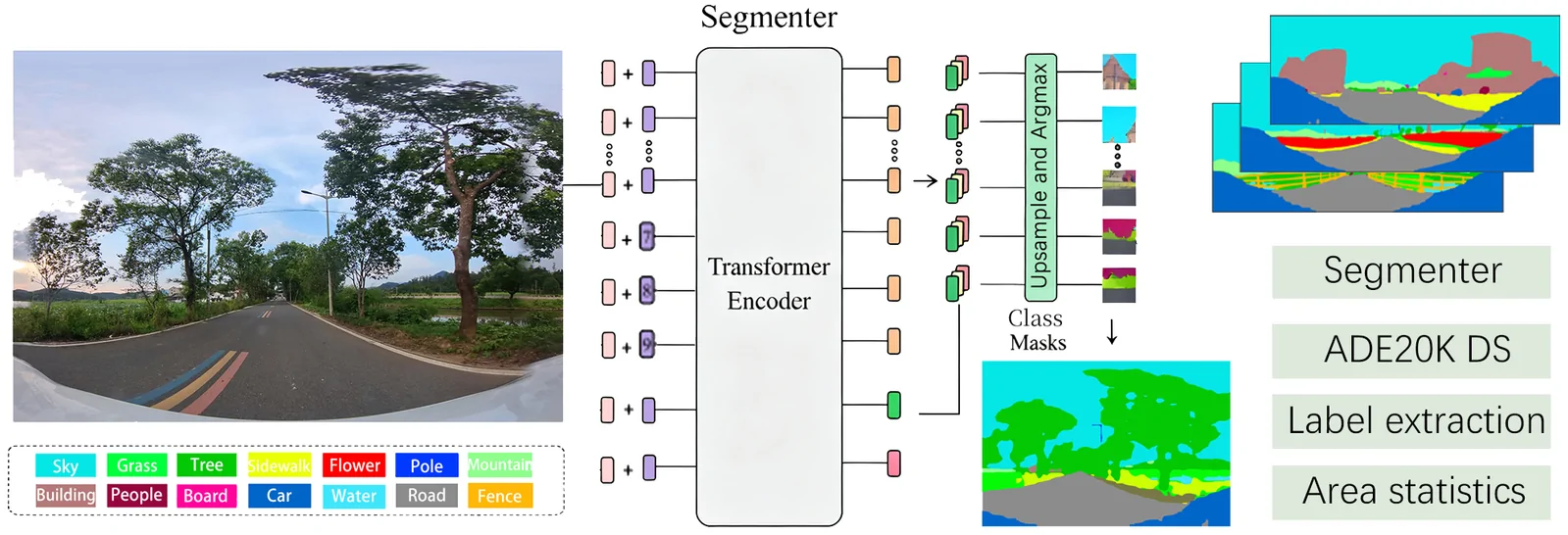
Semantic segmentation using the Segmenter model on the ADE20K dataset.

This study selected the open-source semantic segmentation algorithm library MMSegmentation provided by OpenMMLab. Using the Featurize platform and PyTorch, the model was optimized and applied, with data visualization analysis and image processing carried out using Matplotlib, tqdm, and OpenCV-Python [30,31]. The Segmenter model was trained on the ADE20K dataset for semantic segmentation, enabling efficient and accurate segmentation of panoramic image data from the No. 1 Scenic Road. The identification and segmentation of landscape elements based on image content serve as the technical guarantee for the objective measurement of the scenic road. Based on image recognition technology and binocular visual field images, this study follows three steps (Fig 4): (1) semantic segmentation of different landscape elements, (2) color block extraction for different semantic labels, and (3) pixel-wise traversal calculation of the labeled color blocks to compute the area proportion of each element.

### 2.5. Questionnaire survey and emotional scale

This study employed objective multiple-choice questions to survey respondents’ demographic characteristics and used self-report questionnaires to collect information on their perceptions and emotional states. The formal survey was conducted from March 25 to June 30, 2025, both indoors and outdoors (under predominantly sunny or cloudy weather conditions). Ethical approval was obtained for this study, and all participants provided informed consent prior to participation. The survey was anonymous and voluntary, in accordance with the Declaration of Helsinki. The questionnaire consisted of three sections.

(1) Demographic information, including age, gender, occupation, education level, prior visiting experience, and current emotional state. Considering that scenic roads are primarily traversed by vehicles, and previous research identifies anger, fatigue, stress, confusion, tension, and sadness as critical factors affecting driving safety [32], this study integrated the Positive and Negative Affect Schedule (PANAS) developed by Watson et al. Furthermore, based on the 18 emotional categories classified by Otamendi-Urroz et al. [33] and Quoidbach et al. [34], eight specific emotions were selected following expert consultation and a review of relevant literature. These included four positive emotions (pleasant, excited, calm, relaxed) and four negative emotions (disappointed, irritable, tense, depressed). A 5-point Likert scale, ranging from 1 (not at all) to 5 (extremely strong), was employed for subjective scoring.
(2) Emotional data collection. A total of 131 typical scenes were selected and divided into 13 groups. To ensure the representativeness, each group contained a balanced distribution of scenes from Sections A, B, C, and D, with a requirement that each scene be evaluated by at least 15 volunteers. Following previous controlled VR-based emotion and environmental perception studies [35–37] each scene was evaluated by at least 15 participants, and individual ratings were aggregated into a scene-level mean emotional fluctuation value for MaxEnt modelling. During the experiment, participants wore Samsung Gear VR headsets (SM-R324) for an immersive observation of each scene for a minimum of 20 seconds. Following the observation, volunteers completed an assessment regarding emotional states and preference levels, while also identifying specific environmental elements that triggered emotional fluctuations.
(3) Experiential feedback and behavioral intentions. This part included evaluations of the scene restoration accuracy provided by the VR equipment, participants’ willingness to visit the site in person, and identification of scenes that evoked the most significant emotional responses. By encouraging participants to express their authentic perceptions, this section facilitated a more comprehensive evaluation of the experimental validity.

### 2.6. Calculation of emotional fluctuation value

To quantify emotional changes during landscape experiences, we constructed the Emotional Fluctuation Value (EFV). EFV is derived from the Emotional Nonparametric Relationship Index (ENRI), which follows the calculation method proposed by Garau et al [5]. ENRI is used to assess the emotional relationship between individuals and the landscape, which is categorized as positive (ENRI > 0), negative (ENRI < 0), or polarized (ENRI = 0), and is calculated as shown in Eq (1):


$$\begin{array}{c} ENRI\;=log\left(\;\frac{\sum_{i=\text{1}}^{n}{PositiveEmotionscore}_{i}}{\sum_{i=\text{1}}^{n}{NegativeEmotionscore}_{i}}\;\right) \end{array}$$ (1)

where log represents the logarithmic function, ${\text{Positive Emotion score}}_{\text{i}}$ refers to the rating given by the subject for positive emotions regarding the landscape of the i-th scene, and ${\text{Negative Emotion score}}_{\text{i}}$ refers to the rating for negative emotions in i-th scene.

The Emotional Fluctuation Value (EFV) is calculated by subtracting the initial emotional value from the questionnaire-derived emotional value, in Eq (2):


$$\begin{array}{c} \text{EFV}=\text{ENR}{\text{I}}_{\text{questionnaire }}-\text{ ENR}{\text{I}}_{\text{initial}} \end{array}$$ (2)

where $\text{ENR}{\text{I}}_{\text{questionnaire}}$ denotes the emotional score obtained from the questionnaire, and $\text{ENR}{\text{I}}_{\text{initial}}$ denotes the initial emotional score.

### 2.7. Indicator selection and MaxEnt prediction model

(1) Indicator selection

To systematically capture the environmental features driving emotional fluctuations, we **initially** selected 19 indicators across four dimensions (Table 1) based on established approaches in previous studies [31–33]. The emotional indicators were developed with reference to established frameworks in environmental psychology and landscape perception research [38–40]. Before model construction, correlation tests were conducted on all candidate factors. Variables with Pearson correlation coefficients above 0.8 were removed to reduce multicollinearity, along with those showing zero contribution to emotional prediction.

**Table 1:** A multi-dimensional emotional driving factor system.

Dimension	Indicator	Indicator Description
Nature	Water	The proportion of water area in the image.
	Mountain	The proportion of mountain area in the image.
	Green view index(GVI)	The proportion of all green elements in the image.
	Naturalness	The ratio of natural elements (plants, rocks, mountains, animals, etc.) to grey infrastructure.
	Pastoral landscape index	The combined area proportions of farmland, tea fields, tea mountains, flowers, and flower fields.
	Sky	The proportion of sky area in the image.
Artificial facilities	Road	The proportion of the road area in the image.
	Service facilities	The cumulative proportion of functional and decorative facilities, such as tables, chairs, streetlights, signs, and sculptures.
	Spatial division	The cumulative proportion of elements that disrupt the cohesion of activities and create separated spaces, such as pathways, walls, stairs, and columns.
Visual perception	Spatial enclosure	Measures the degree to which vertical elements (trees, buildings, walls, etc.) enclose or open up the horizontal space, reflecting the sense of enclosure in the space.
	Visual field analysis	Quantitative analysis of the visible range along the scenic road using ArcGIS 10.8, based on DEM or 3D terrain models, employing line-of-sight algorithms to assess visibility obstructions caused by terrain or objects.
	Color richness	Refers to the diversity, complexity, and distribution balance of colors in the image.
	Visual entropy	Visual entropy, an indicator of visual information complexity, is calculated in MATLAB through grayscale enhancement, regional segmentation, and area calculation. It reflects the diversity and disorder of elements like brightness and texture.
	Skyline complexity	The irregularity and dramatic changes in the boundary line between the sky and the landscape elements or background.
Environmental background	NDVI	NDVI extracted from Landsat 8 OLI satellite imagery.
	DEM	DEM data from the China Geospatial Data Cloud (https://www.gscloud.cn) platform.
	Land use	Based on Google remote sensing satellite map data.
	Slope	Calculated using elevation data with ArcGIS 10.8.
	Aspect	Calculated based on slope data using ArcGIS 10.8.

(2) MaxEnt Prediction Model

The Maximum Entropy (MaxEnt) model is a machine learning algorithm that predicts the most uniform probability distribution under known environmental constraints, optimizing predictions with incomplete data [41]. The model uses known event locations and their corresponding environmental variables to predict the probability of event occurrence across unsampled locations. Originally developed for species distribution modelling, MaxEnt has been widely extended to other presence-only spatial prediction tasks, including cultural ecosystem services, recreational suitability, environmental risk assessment, and other human–environment interaction studies [42–45]. Its ability to capture complex nonlinear relationships from limited presence samples makes it particularly suitable for modelling spatial patterns of human emotional responses.

A 2-km buffer along the No. 1 Scenic Road was defined as the MaxEnt calibration area to represent the road-centered landscape context relevant to scenic-road travel, following a previous landscape study [46]. All environmental raster layers were clipped to this buffer and aligned to the same resolution and coordinate system. Background data were generated from valid raster cells within the buffer, while emotional sampling points along the road were used as presence data. Then randomly selected 75% of the emotional sampling points as the training set and the remaining 25% as the test set. The model was run 10 times to evaluate its stability. The model’s prediction accuracy was assessed using the Area Under the Curve (AUC) value, with values closer to 1 indicating higher accuracy [47].

Beyond high predictive accuracy, the MaxEnt (Version 3.4.3) model offers excellent interpretability by outputting factor contribution rates and response curves. This allows for the precise identification of key landscape drivers and their nonlinear thresholds, providing direct quantitative support for refined scenic road management.

### 2.8. Local Moran’s I

Local spatial autocorrelation was analyzed using the Local Moran’s I index to investigate whether positive and negative emotional fluctuations exhibit spatial dependence and spatial heterogeneity [48], as shown in the following formula:


$$\begin{array}{c} {\text{I}}_{\text{i}}=\frac{({\text{x}}_{\text{i}}-\overset{―}{\text{x}})}{{\text{S}}^{\text{2}}}\sum_{\text{j}=\text{1},\text{j≠i}}^{\text{n}}{\text{W}}_{\text{ij}}({\text{x}}_{\text{j}}-\overset{―}{\text{x}}) \end{array}$$ (3)

$$\begin{array}{c} {\text{S}}^{\text{2}}=\frac{\sum_{\text{j}=\text{1},\text{j≠i}}^{\text{n}}{({\text{x}}_{\text{j}}-\overset{―}{\text{x}})}^{\text{2}}}{\text{n}-\text{1}} \end{array}$$ (4)

In Eqs (3) and (4), ${\text{I}}_{\text{i}}$ represents the Local Moran’s I value for spatial unit i; ${\text{x}}_{\text{i}}$ represents the Local Moran’s I value for spatial unit $\text{i};\;\bar{\text{x}}$ represents the mean emotional fluctuation value of all spatial units; ${\text{x}}_{\text{j}}$ represents the emotional fluctuation value of another spatial unit j (j≠i); ${\text{W}}_{\text{ij}}$ is the element of the spatial weight matrix, characterizing the spatial proximity relationship between units i and j (this study uses a distance-based weight setting); n is the total number of spatial units; ${\text{S}}^{\text{2}}$ is the sample variance of the emotional fluctuation values.

## 3. Results

### 3.1. Analysis of emotional questionnaire results

This study collected a total of 2,111 valid questionnaires from 209 respondents. The sample had a relatively balanced gender ratio (46.9% male, 53.1% female) with the majority of respondents aged 18–35 years (52.6%). Although only 37.3% of participants had visited the scenic road in person, 82.5% expressed an intention to visit after experiencing the VR simulation. Furthermore, 91.0% of participants rated the scene reproduction as “relatively realistic” to “highly realistic”, indicating that the immersive VR visual scenes were able to reliably reproduce the scenic road environment and provide a credible situational basis for capturing visitors’ emotional responses.

The overall emotional fluctuation results showed that positive emotions dominated the visitor experience (Table 2). Positive emotional fluctuations accounted for 56.23% of the samples (n = 1,187), significantly higher than negative emotional fluctuations (38.37%, n = 810) and instances with no emotional fluctuations (5.40%, n = 114). The overall range of emotional fluctuation values was quite broad, ranging from −1.255 to 1.376, reflecting significant differences in emotional experiences across different scenes. However, from an overall statistical perspective, the mean of the emotional fluctuations approached neutral (Mean = 0.034, variance = 0.3640), indicating that, at the overall level, positive and negative emotions somewhat offset each other spatially, with the overall experience still slightly leaning towards positive emotions.

**Table 2:** Statistics of emotional fluctuation results in the questionnaire.

Types of emotional fluctuations	Sample size(copy)	Proportion(%)	Fluctuation range[min,max]
Positive fluctuation	1187	56.23	[0.007,1.376]
Negative fluctuation	810	38.37	[-1.255,-0.007]
no fluctuation	114	5.40	[0,0]

**Fig 5:**
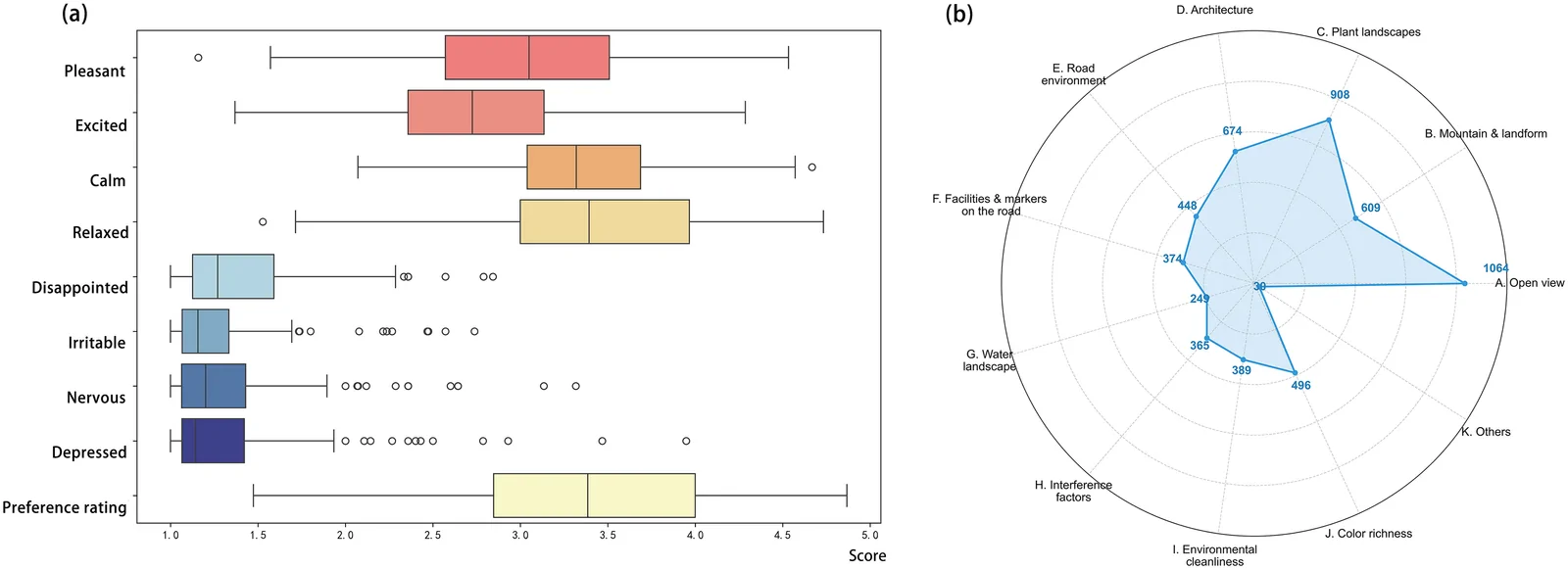
Analysis of questionnaire results. (a) The results of emotions and preference. (b) The proportion of factors that trigger emotional fluctuations.

At the specific emotional dimension level, “relaxed” (Mean = 3.42) and “calm” (Mean = 3.33) were the most prominent positive emotional states (Fig 5a), reflecting that the scenic road primarily provides a soothing and tranquil emotional experience. Visitors’ general level of fondness for the environment was relatively high (Mean = 3.38), which was closely correlated with the intensity of these positive emotional experiences, confirming the strong connection between emotional experiences and environmental preferences.

Inductive analysis based on open-ended questions further revealed the primary environmental factors eliciting emotional fluctuations (Fig 5b). Among these, the degree of open view (n = 1064) and plant landscapes (n = 908) were the most prominent environmental elements triggering emotional responses. This was followed by architectural character (n = 674) and mountains and landforms (n = 609). In contrast, detailed elements such as facilities and colors were mentioned less frequently. However, they still exerted an influence on emotional experiences in specific segments. Notably, disturbance factors (n = 365) were predominantly associated with negative emotional experiences in respondents’ descriptions, revealing their important role in explaining differences in emotional responses along the scenic road.

### 3.2. Overview of scenic road landscape features

Based on the semantic segmentation analysis of 614 panoramic photos, the overall composition and spatial differentiation patterns of the landscape elements along the No. 1 Scenic Road were characterized. Overall, the landscape is dominated by natural elements, with a relatively low proportion of artificial features, reflecting a clear natural characteristic. Among these, the sky (mean = 40.12%) accounts for the highest proportion of landscape elements, indicating a high degree of visual openness along the road. GVI (mean = 0.28) and high naturalness (mean = 2.28) suggest a high level of vegetation coverage. In contrast, the proportion of artificial elements is overall low (mean = 2.01%), highlighting the weak degree of human intervention in the landscape along the scenic road.

**Fig 6:**
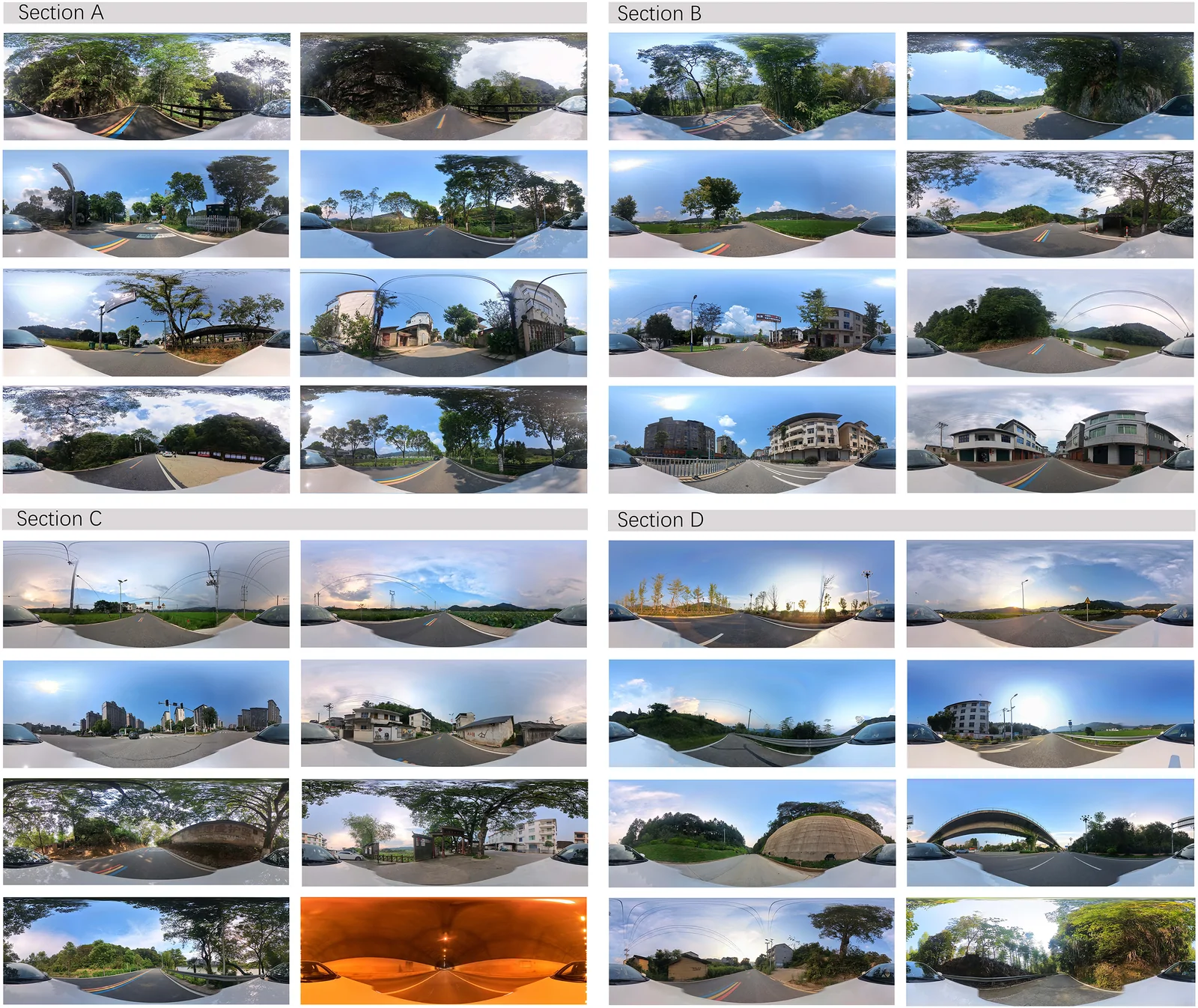
Representative landscapes of each section.

Although natural elements dominate overall, the landscape along the road exhibits significant spatial heterogeneity. The spatial differentiation primarily unfolds along a notable altitude gradient (153.85 to 1,166.47 meters), forming a continuous landscape structure sequence between low and high elevations (Table 3 and Fig 6).

**Table 3:** Main landscape features and section descriptions of the No. 1 Scenic Road.

Section	Starting and Ending Point	Dominant Landscape Character
Section A	Nan Yuanling – Huangkeng Town	High-elevation dense forest canyon with strong enclosure and wilderness immersion
Section B	Huangkeng Town – Jukou Town	Mid-low elevation, urban-rural transition landscape.
Section C	Jukou Town – Wufu Town	Low-elevation open valley with concentrated historical towns and higher human facilities
Section D	Wufu Town – Nan Yuanling	Mid-high elevation grand vista with open sky and alpine meadows

Specifically, the high-altitude section (Section A, average elevation = 458 m) shows higher values in the GVI, naturalness, and space enclosure, with a high level of vegetation coverage. The landscape is primarily composed of forests and mountains. As the elevation decreases, Section B exhibits a high degree of landscape element mixing, with alternating patterns of farmland, forests, and settlements, while the visibility of water bodies reaches a relatively high level. The lowest elevation section, Section C shows a higher proportion of visible sky (48.1%) and a relatively higher proportion of artificial elements, with an open valley and cultural landscape dominating the landscape structure. As elevation rises further, Section D maintains a high degree of visual openness while exhibiting a richer color index, forming a landscape combination with significant spatial distinctiveness.

Overall, the landscape elements along the No. 1 Scenic Road present a clear spatial differentiation pattern along the altitude, transitioning from nature-dominated landscapes at higher altitudes to mixed and cultural landscapes at lower altitude. This continuous and orderly landscape heterogeneity provides an objective environmental background for subsequent analysis of emotional spatial distribution and driving factors.

### 3.3. MaxEnt model-based emotional prediction map and spatial distribution characteristics

#### 3.3.1. Predictive performance of MaxEnt.

**Fig 7:**
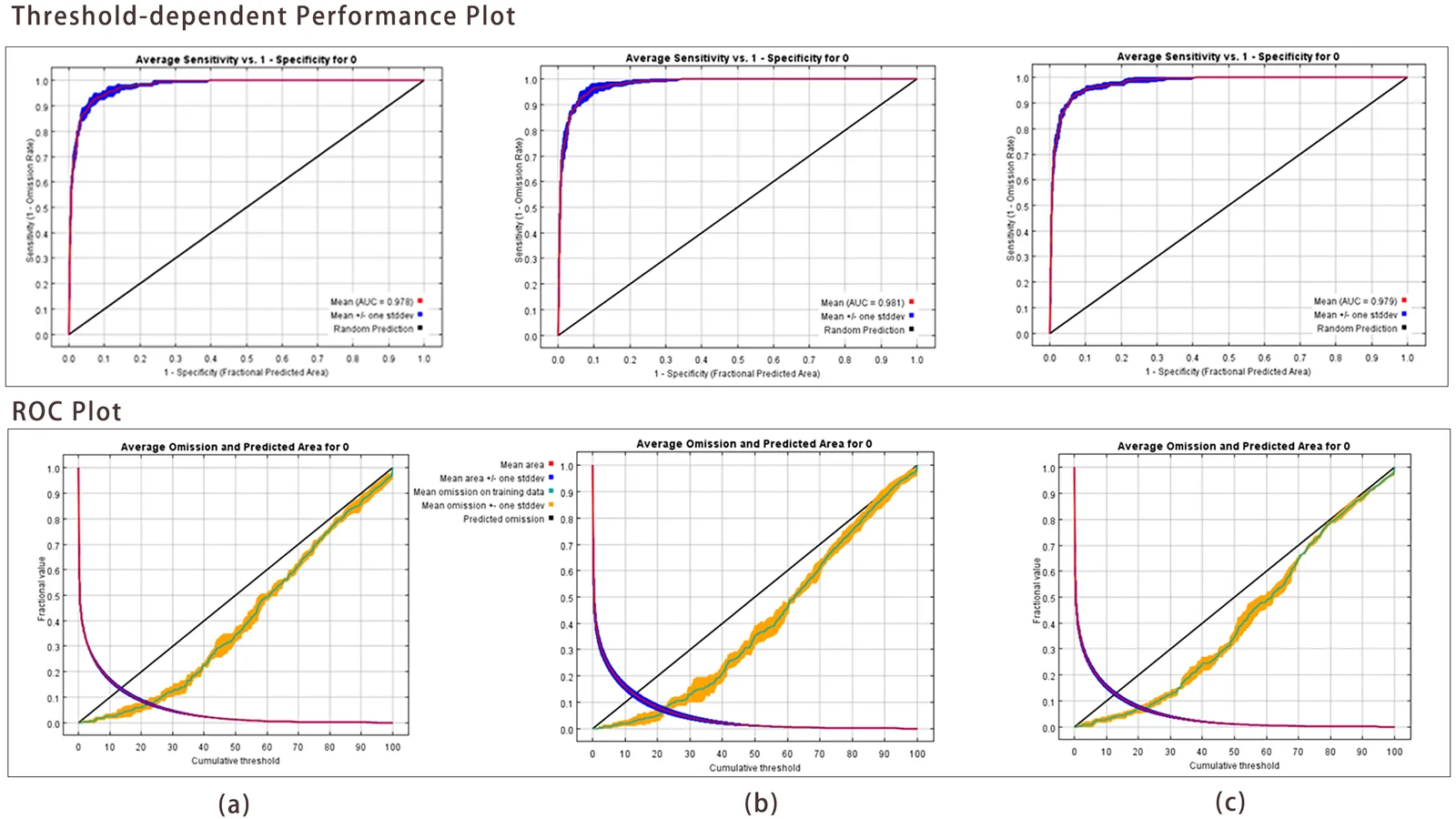
Predictive performance of the MaxEnt model. (a) Positive, (b) Negative, and (c) All types of emotional fluctuations.

After controlling for correlations among input variables, the MaxEnt model demonstrated high stability and accuracy in predicting the spatial distribution of emotional fluctuations. The results from running the model 10 times indicated that emotional fluctuations exhibit good predictability (Fig 7). Specifically, the average AUC value for positive emotional fluctuations was 0.978, for negative emotional fluctuations was 0.981, and for all types of fluctuations was 0.979. All of these values were significantly above the high-performance threshold of 0.900, indicating that the model can effectively characterize the spatial probability of emotional fluctuations.

These results suggest that emotional fluctuations are not randomly distributed; rather they can form stable probabilistic structures under the constraints of environmental variables. This provides a reliable foundation for subsequent analysis of emotional spatial distribution patterns.

#### 3.3.2. Overall spatial distribution characteristics of emotional fluctuation zone.

To characterize the overall spatial distribution structure of emotional fluctuations, the emotional occurrence probability (P) produced by the MaxEnt model is used. The Natural Breaks classification method is applied to classify the predicted results, dividing the probabilities of emotional fluctuations into five categories: Low (0 ≤ P < 0.0626), Low-Medium (0.0626 ≤ P < 0.168), Medium (0.168 ≤ P < 0.321), Medium-High (0.321 ≤ P < 0.540), and High (0.540 ≤ P < 1) fluctuation zone. Based on this classification, a spatial distribution map of emotional fluctuations is generated.

Overall, emotional fluctuations along the scenic road exhibit significant spatial differentiation. The Medium-High fluctuation zone occupies a large proportion of the area (43.34%, 120.96 km), indicating that emotional fluctuations are not scattered but rather form continuous high-value segments within a certain range. Meanwhile, the Low fluctuation zone accounts for 27.29% of the area (76.16 km), showing a relatively concentrated spatial distribution.

**Fig 8:**
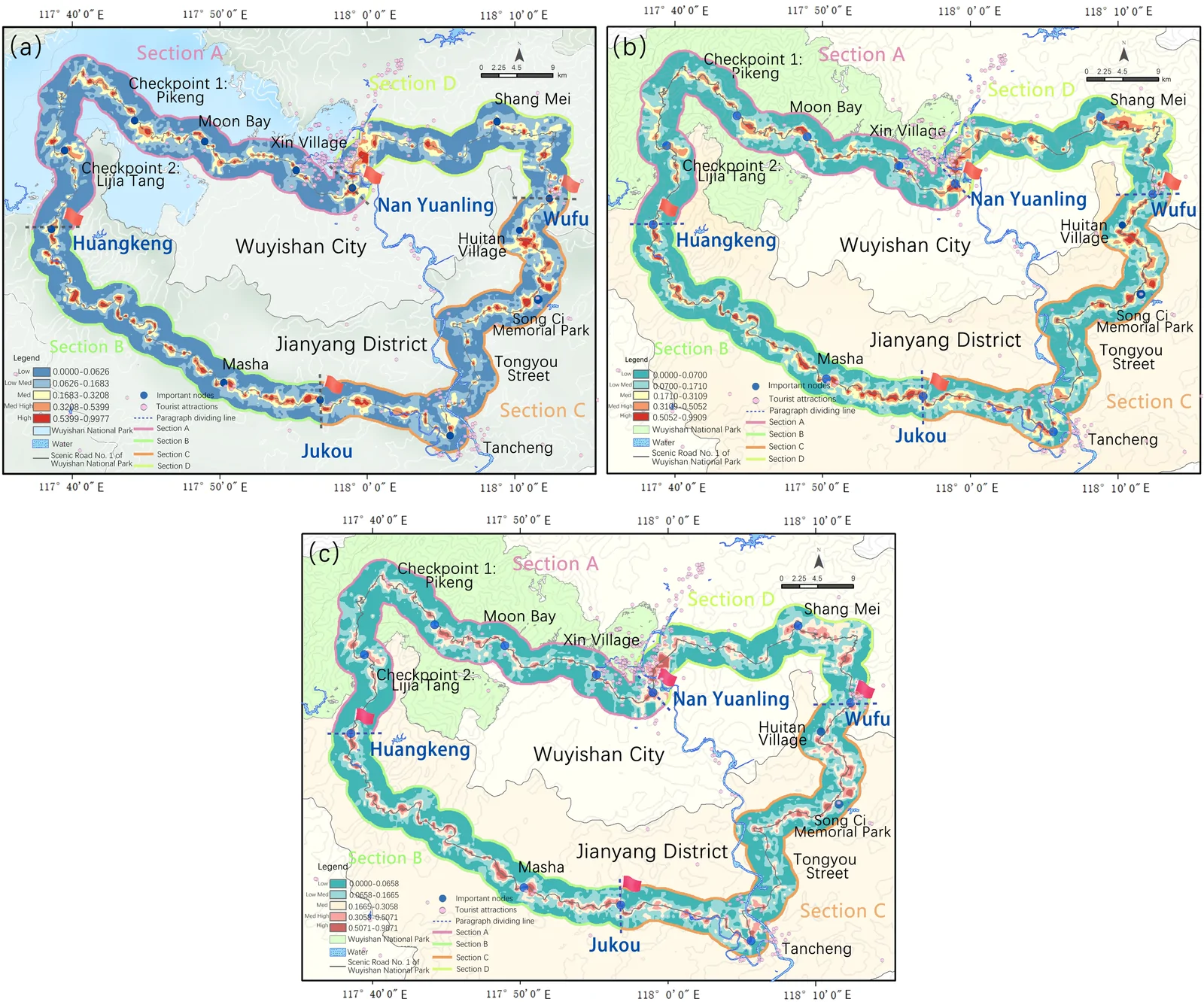
Spatial distribution of predicted emotional fluctuation probability. (a) All types, (b) Positive, and (c) Negative fluctuations.

Regarding spatial configuration (Fig 8), High fluctuation zone is distributed discontinuously yet form interconnected clusters along the main route, particularly around specific nodes. In contrast, Low fluctuation zone shows more continuous distribution patterns in some sections. Overall, emotional fluctuations along the scenic road display a spatial structure characterized by the interspersed distribution of Low, Medium, and High fluctuation zones.

#### 3.3.3. Emotional spatial distribution characteristics of the main scenic road and buffer zone.

To explore the differences in the spatial structure of emotional distribution, this study analyzes emotional fluctuations at two scales: the linear scale of the main scenic road and the 2-km buffer zone on both sides of the scenic road. The prediction results show that the high-value emotional fluctuation areas exhibit significantly different spatial distribution patterns at the two scales. Along the main scenic road, they are primarily linearly concentrated, while in the buffer zone, they are more dispersed and patch-like.

At the scale of the main scenic road, there are significant differences in emotional fluctuation levels across different sections (Table 4). Section B has the highest mean emotional fluctuation with the greatest variance (mean = 0.3977, variance = 0.0516), indicating that this section stands out in terms of both the intensity and the range of emotional fluctuations. Section C has the second-highest emotional fluctuation level (mean = 0.3493). In contrast, Section A and D display relatively lower mean fluctuation values with gentler variation amplitudes, presenting a stratified difference among the sections along the route. When the analysis scale is expanded to the 2-km buffer zone on both sides of the scenic road, the spatial pattern of emotional fluctuations changes significantly. Compared to the main scenic road scale, the mean emotional fluctuations in the buffer zone are generally much lower, only about 25%−33% of those at the main scenic road scale. At the buffer zone scale, Section C has the highest mean emotional prediction (mean = 0.1183), followed by Section D (mean = 0.1174). The mean values for the buffer zone of Section B (mean = 0.1020) and Section A (mean = 0.0937) are relatively lower, demonstrating a ranking order different from that observed at the main road scale.

**Table 4:** Predicted emotional fluctuation values along the main road and within the 2-km buffer zone.

Scale	The Main Road				The 2-km Buffer Zone			
Section	A	B	C	D	A	B	C	D
Mean	0.3013	0.3977	0.3493	0.2777	0.0937	0.1020	0.1183	0.1174
Max	0.9493	0.9977	0.9941	0.9667	0.9855	1.0000	0.9999	0.9993
Min	0.0002	0.0020	0.0266	0.0104	0.0000	0.0000	0.0001	0.0000
Variance	0.0364	0.0516	0.0444	0.0404	0.0188	0.0282	0.0243	0.0230
Median	0.2612	0.3806	0.3105	0.2306	0.0440	0.0381	0.0700	0.0662

#### 3.3.4. Spatial distribution characteristics of positive and negative emotional fluctuations.

To compare the spatial distribution differences between different emotional directions, we separately mapped the spatial patterns of positive and negative emotional fluctuations (Fig 8b and Fig 8c) and conducted a comparative analysis of their spatial characteristics. The results show that positive and negative emotional fluctuations exhibit differentiated spatial distribution characteristics across different sections.

For positive emotional fluctuations, the high-value areas are mainly concentrated in certain specific sections. Among them, Sections C (Mean = 0.1291) and D (Mean = 0.1293) have the highest mean positive emotional predictions, showing relatively prominent positive emotional fluctuation levels. In contrast, the mean positive emotional values for Sections A and B are relatively low. The high-value areas for positive emotions exhibit a certain clustering pattern in space, corresponding to several key areas along the scenic road.

The spatial distribution of negative emotional fluctuations presents different characteristics. The mean negative emotional prediction reaches its highest in Section B (Mean = 0.1398), with its negative emotional level being higher than the corresponding positive emotional value for that section, indicating that this section stands out in terms of negative emotional fluctuations. Sections C and D also show relatively high negative emotional values, while Section A has relatively low negative emotional fluctuations, showing clear differences between the road sections.

**Fig 9:**
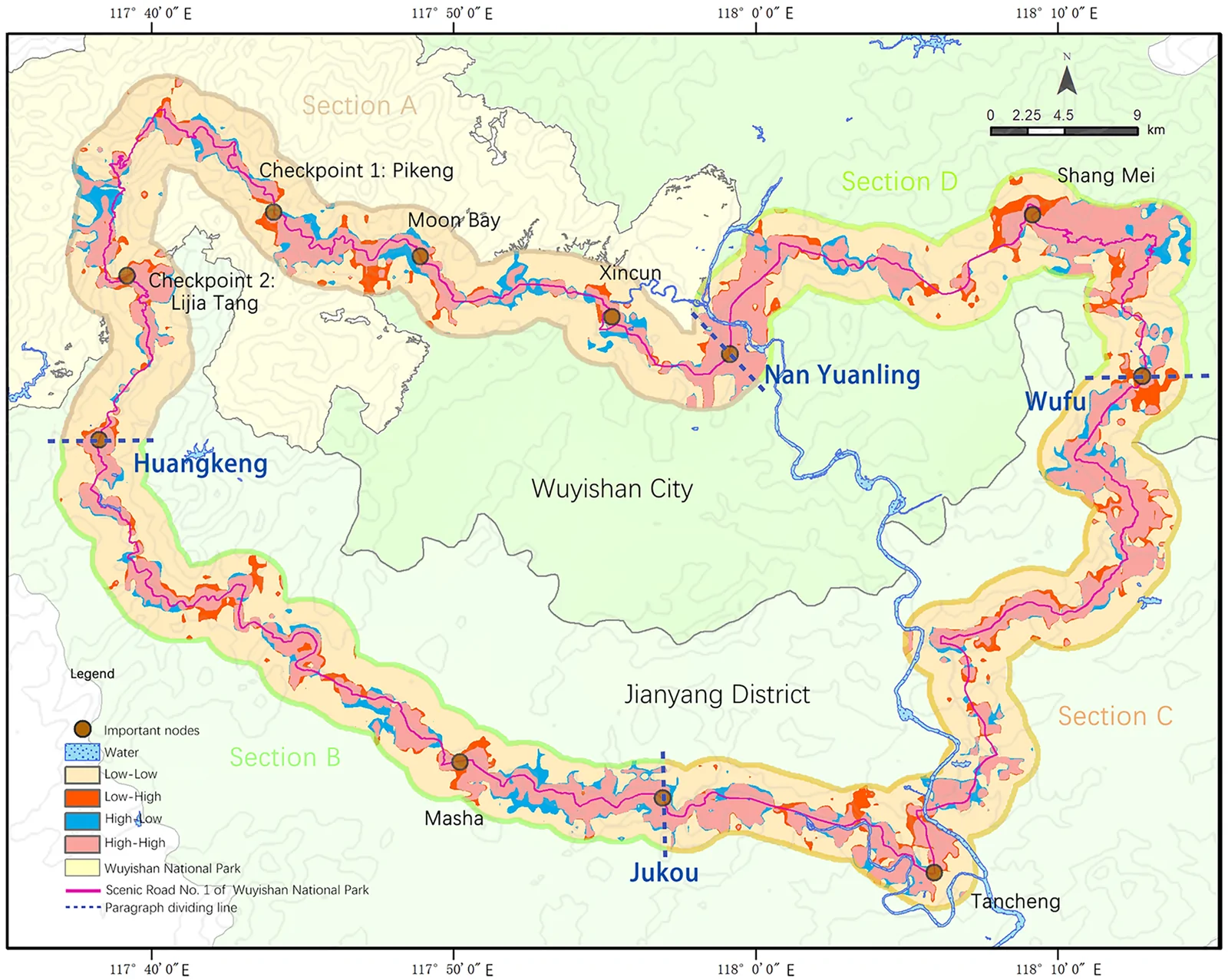
Local Moran’s I distribution results for positive and negative fluctuations.

Further spatial autocorrelation analysis indicates that both positive and negative emotional fluctuations show significant clustering patterns, forming a pattern of co-occurrence between positive and negative emotions (Fig 9). The local Moran’s I results reveal that the “Low Positive - Low Negative” clustering area is the most widespread, covering an area of 540.45 km^2^, accounting for 61.26% of the study area; the “High Positive - High Negative” clustering area covers 255.20 km^2^, accounting for 28.93%. The areas of “High Positive - Low Negative” and “Low Positive - High Negative” are smaller, accounting for 4.83% and 4.97%, respectively. Overall, the “High Positive - High Negative” and “Low Positive - Low Negative” clustering areas dominate, together accounting for 90.20%.

In terms of spatial distribution, the “High Positive - High Negative” clustering area is mainly distributed along the main scenic road, while the “Low Positive - Low Negative” clustering area is concentrated in the outer buffer zone, far from the main road. The “High Positive - Low Negative” and “Low Positive - High Negative” areas are mostly located in between, forming a transitional zone. The results indicate that positive and negative emotional fluctuations exhibit clear clustering structures in space, rather than random distribution.

### 3.4. Driving factors and nonlinear thresholds influencing emotional spatial distribution

**Fig 10:**
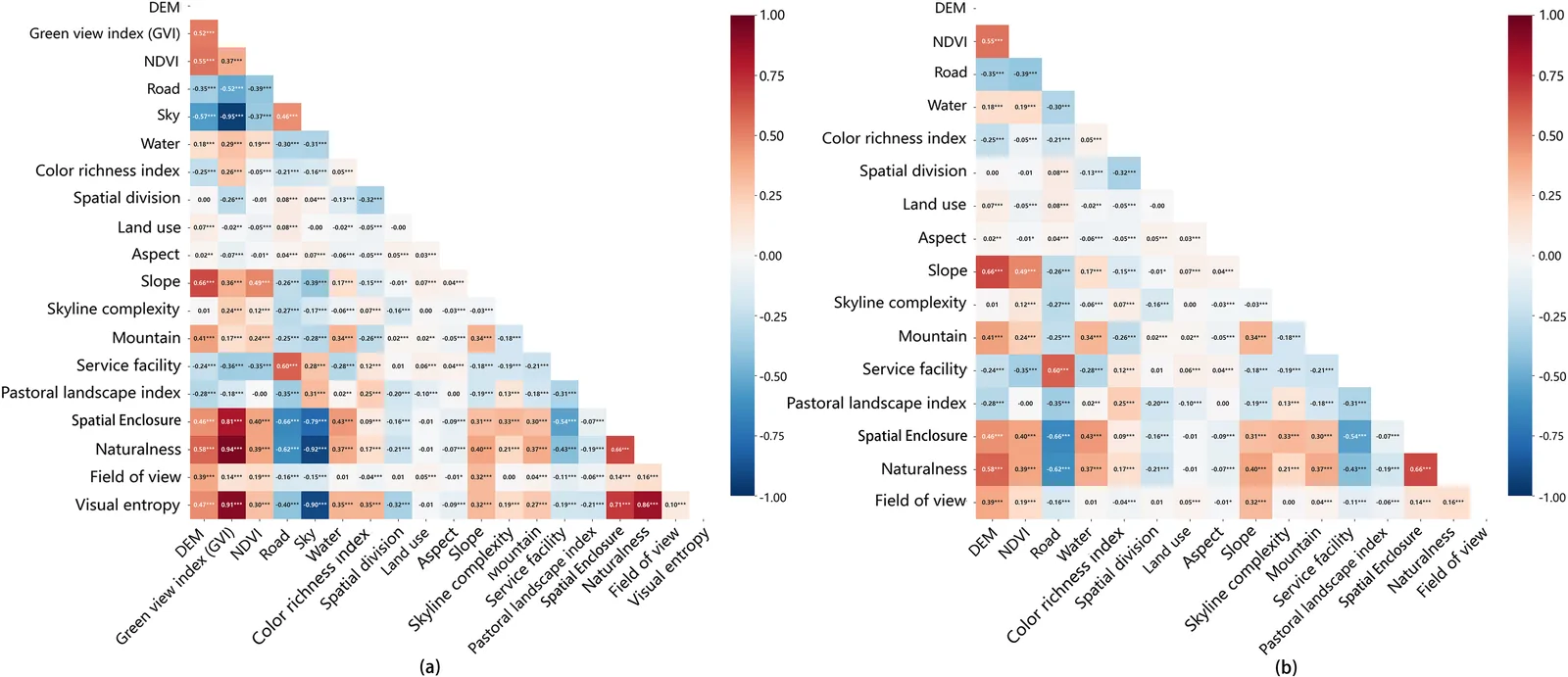
Pearson correlation analysis of driving factors. (a) Initial driving factors. (b) Driving factors with strong correlations removed.

After screening the candidate environmental variables, 16 driving factors were included in the model, primarily including variables such as visual field, pastoral landscape index, and naturalness. In contrast, variables such as sky, GVI, and visual entropy were excluded from the final spatial analysis of emotions due to issues of high multicollinearity (Pearson correlation coefficient > 0.8) (Fig 10).

#### 3.4.1. Relative contribution rate of emotional driving factors.

**Fig 11:**
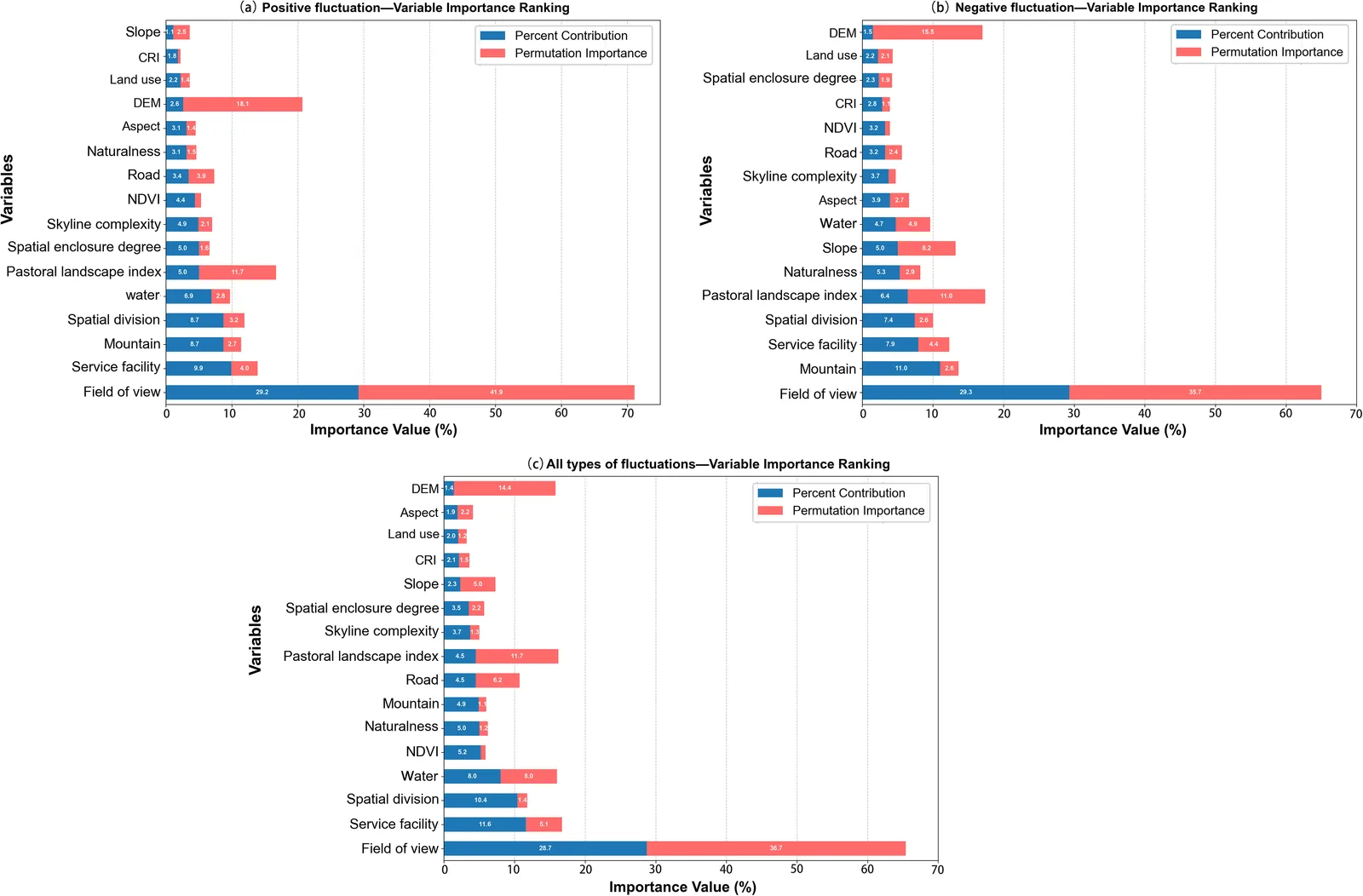
Contribution rate of MaxEnt factors. (a) Positive, (b) Negative, and (c) All types of fluctuations.

The results of the MaxEnt model interpretation highlight the key driving factors (Fig 11). These factors show significant differences in how various environmental variables influence emotional fluctuations along the scenic road. Among the three fluctuation models, visual field is the most dominant driving factor for emotional fluctuations (Average Percent Contribution, APC = 29.1%), and it has the highest independent explanatory power in the jackknife test. In addition to visual field, service facilities (APC = 9.8%), mountains (APC = 9.5%), spatial division (APC = 8.3%), and water (APC = 6.5%) also show relatively high contribution rates, making them important factors influencing the spatial distribution of emotions. Although elevation has a lower direct contribution, it has a high Average Permutation Importance (API = 16.0%), indicating its significant role in emotional spatial distribution.

Further comparison of the model results for different emotional directions reveals clear differences in the relative importance of driving factors for positive and negative emotional fluctuations. In the negative emotional fluctuation model, the contribution of slope significantly increases compared to the positive model, and the relative contribution of naturalness also rises. In contrast, in the positive emotional fluctuation model, the contribution of water significantly increases. A closer analysis of the positive emotional hot areas shows that the visual proportion of water exhibits a clear low-threshold characteristic: in 95.59% of the positive emotional hots areas, the visual proportion of water is below 0.35%, indicating that the role of water in positive emotions is mainly related to their visual visibility rather than their actual size or area.

#### 3.4.2. Nonlinear threshold characteristics of emotional driving factors.

**Fig 12:**
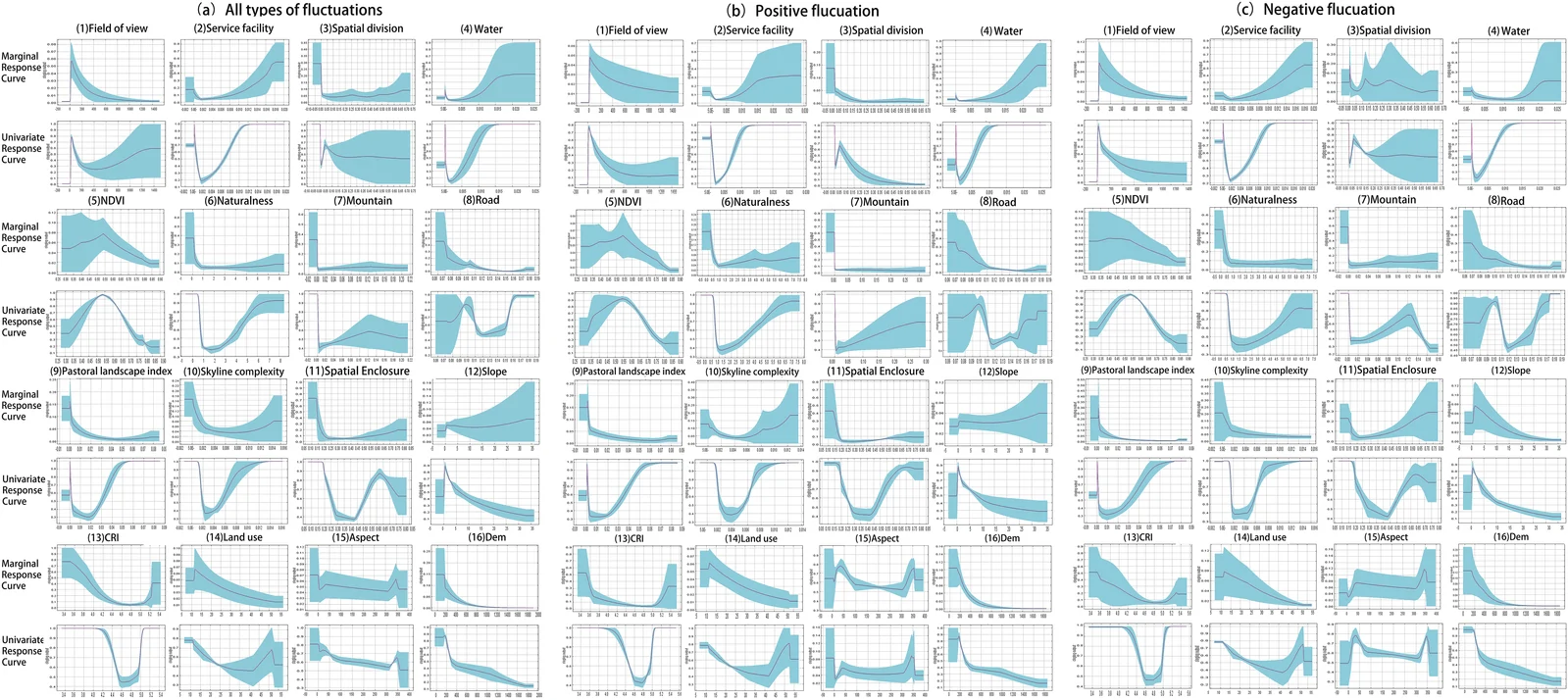
Response curves of driving factors. (a) All types, (b) Positive, and (c) Negative fluctuations.

The response curve analysis of the MaxEnt model (Fig 12) indicates that various emotional driving factors have significant nonlinear effects on the spatial distribution of emotional fluctuations. Different factors show the most prominent influence on emotional fluctuations within specific value ranges, while the emotional response intensity significantly decreases below or above these ranges, exhibiting clear threshold effects. Some fluctuations are observed at the extreme ends of several response curves. These fluctuations are likely attributable to the limited representation of samples under extreme environmental conditions, which may increase uncertainty in model estimation and may represent potential model artifacts at the tails of the response curves. Accordingly, the interpretations presented in this study primarily focus on the response trends within the main environmental ranges where the sample distribution is denser.

Among the natural landscape elements, water and the pastoral landscape index significantly enhance positive emotions and reduce negative emotions under relatively low proportions. Specifically, water visibility shows a higher positive emotional response when the visibility is within the range of 1% to 2.5%. The pastoral landscape index significantly enhances emotional fluctuations when its proportion exceeds 2%. Mountain shows differentiated threshold intervals for different types of emotional fluctuations: positive emotional fluctuations show a continuous increase when visibility exceeds 5%, while all types of fluctuations (4% − 14%) and negative emotional fluctuations (4% − 12%) peak within specific ranges, with responses diminishing beyond these ranges.

Regarding the artificial landscape elements, the proportion of service facilities is significantly correlated with positive emotional fluctuations within the 10% − 20% range, with positive emotional responses increasing as the proportion rises. Road and spatial division exhibit multi-peak fluctuations in the Univariate Response Curve, revealing a multi-level mechanism of emotional responses. Road shows different response mechanisms across the three fluctuation types: positive and all types of fluctuations peak at approximately 10%, 14% and 16%. Within 11%−15%, the impact on emotions is relatively positive and stable. However, both narrow (8% − 10%) or excessively wide (>15%) roads are associated with a marked increase in negative emotional fluctuations. The influence of spatial division is complex: low spatial division values favor positive emotional responses, while negative emotions show local peaks at multiple value intervals (around 0%, 15%, and 40%).

In terms of visual and spatial perception, visual field indicates that emotional hotspots consistently maintain identifiable effective values, suggesting that high-intensity emotional areas require a certain degree of visual exposure. Skyline complexity shows a significant positive correlation with positive emotional fluctuations within a moderate range; richer contour variations tend to yield more pronounced positive emotions. Color richness also exhibits distinct nonlinear characteristics, with emotional fluctuations becoming more significant at the lower (<3.4) and higher (>5.0) ranges. Spatial enclosure similarly shows a range-based response characteristic, where mid-to-high enclosure levels (0.45 to 0.8) enhance emotional fluctuations, with negative emotional fluctuations showing a higher magnitude of change.

Among the macro-environmental factors, the NDVI in the 0.5–0.6 range corresponds to higher emotional responses, while negative emotional fluctuations increase when it is below 0.3 DEM shows stronger emotional responses at low-to-medium altitudes (<400 m) but gradually weakens as elevation increases. Slope is more closely associated with negative emotions in the 0°-5° range, and emotional fluctuations stabilize after exceeding 20°. There are also differences between slopes facing different directions, with sunny slopes generally corresponding to higher positive emotional responses than shady slopes. Analysis of land use types shows that emotional hotspots are relatively concentrated in natural-agricultural mixed landscapes, such as paddy fields (31.09%) and forested areas (27.26%).

## 4. Discussion

### 4.1. Hierarchical spatial structure of emotional fluctuations

Existing emotional mapping studies have mostly focused on relatively static areal spaces, such as urban green spaces, parks, or protected areas [5,49]. In these studies, emotional distribution is often represented as point or area-based clustering, emphasizing emotional responses at specific locations (e.g., entrances, nodes, or viewpoints) [50,51], with limited attention given to the fluctuations in emotion. However, as a continuous linear recreational space, the emotional experience of a scenic road is not based on static stops but relies on the ongoing dynamic process of movement, which gives rise to significant fluidity and path dependency in emotional responses. This study extends the emotional mapping framework to the linear scenic road context, revealing a corridor-like clustering of emotional fluctuations along the road, as opposed to the area-based diffusion patterns commonly observed in traditional emotional mapping studies.

This study identifies a hierarchical spatial structure of emotional fluctuations along the scenic road, which differs fundamentally from traditional area-based studies. The emotional distribution follows a clear three-tiered pattern: the core scenic road, the 500-meter buffer zone surrounding it, and the 2-km outer buffer zone. Specifically, at the level of the main scenic road, emotional fluctuations are highly dependent on the visual corridor and the sequence of landscapes encountered during movement, exhibiting significant path dependency characteristics. Within the approximately 500-meter buffer zone surrounding the road, emotional distribution enters a transition area centered on the road, maintaining a strong connection to the path while beginning to expand into the surrounding space. As the lateral distance increases, emotional fluctuations rapidly weaken, presenting a path-dominated, linear clustering pattern. When the spatial scale is further expanded to the 2-km buffer zone, emotional distribution gradually moves away from direct control by the road and begins to resemble the area-based distribution pattern commonly seen in traditional emotional mapping studies. While the main road serves as the central conduit for high-intensity, curated landscape encounters, the surrounding buffer zone functions differently. Its landscape heterogeneity provides a foundational ambiance that, although essential, yields less concentrated emotional salience than the primary travel route. This finding suggests that the emotional spatial distribution of scenic roads is not a simple linear or area-based structure, but rather a spatial structure with significant scale dependence and a clear hierarchical organization. This hierarchical structure provides a new perspective for understanding the spatial organization of emotional experiences in linear recreational spaces.

### 4.2. Coexistence and spatial heterogeneity of positive and negative emotions

Existing research often relies on a simplified assumption that positive and negative emotions are spatially exclusive, with spatial emotions being dominated by one type of emotion. However, the analysis of the scenic road as a linear recreational space reveals that positive and negative emotions are not simply antithetical. Instead, they exhibit significant coexistence in certain core sections, highlighting the inherent complexity of emotional experiences in linear recreational spaces.

The coexistence of positive and negative emotions is closely linked to the dual nature of the landscape environment. On one hand, distinctive landscape elements and clear, readable structures reduce cognitive load and trigger positive emotions. This aligns with Attention Restoration Theory’s expectations for open natural settings [52,53]. On the other hand, these spaces have high information density and strong emotional intensity, making them more sensitive to artificial disturbances or visual obstructions. As a result, negative emotions can be activated at the same time [54]. Therefore, emotional hotspot areas are not spaces characterized by a single emotional attribute, but rather complex emotional spaces that simultaneously possess the potential to evoke both positive and negative emotions.

At larger spatial scales, positive and negative emotions gradually exhibit distinct spatial heterogeneity. This pattern arises from uneven environmental gradients and the sequential character of linear spaces. Specifically, environments with open views, clear spatial structures and high readability are more conducive to reducing cognitive load and consistently stimulating positive emotions. In contrast, spatial settings with complex landscape elements, frequent transitions and a lack of sequential order are prone to information overload and cognitive fatigue, increasing the probability of negative emotions. In linear recreational spaces, emotional experiences are not only related to landscape diversity but are also largely dependent on the organization and rhythm of landscape information, which is consistent with the views of Jiang et al [55]. Moreover, the path-dependent nature of linear spaces amplifies these gradients, causing emotions to cluster strongly in core areas and fade outward, resulting in distinct spatial heterogeneity. It should be noted that the present analysis was conducted at the spatial pattern level. Therefore, the observed coexistence does not necessarily reflect emotional ambivalence or other psychological mechanisms at the individual level. Future studies incorporating individual-level emotional data are needed to further verify these relationships.

### 4.3. Driving factors and nonlinear threshold mechanisms of emotional fluctuations

The results indicate that emotional responses in linear recreational spaces are not the result of a linear accumulation of a single landscape element. Instead, they are influenced by multiple driving factors, exhibiting significant nonlinear and threshold characteristics. Among these, the visual field is the necessary condition for emotional arousal. An open visual field satisfies the inherent human need to monitor and assess the surrounding environment, bringing a sense of security, control, relaxation, and pleasure [56], thereby providing a spatial foundation for emotional arousal. It is important to note that visual elements are not simply better in greater quantities. The complexity of the skyline and color richness can both effectively enhance emotions, but only when in moderate or extreme states, revealing that visual stimuli in linear recreational spaces are more dependent on contrast and variation, rather than continuous accumulation.

The emotional value of natural landscapes lies in the balanced configuration between openness and enclosure, natural abundance and visual permeability, rather than simply maximizing the amount of greenery. Water exhibits a strong positive emotional effect even at relatively low visual proportions. Their effect primarily stems from their role as visual focal points and natural symbols, rather than their size. This finding is consistent with numerous studies confirming the positive psychological benefits of blue spaces [57,58]. In contrast, the emotional effects of mountains and vegetation are more range-dependent. Moderate mountain visibility and vegetation density provide stable visual focal points and a sense of environmental shelter [59]. However, when these elements dominate the visual field, the landscape tends to become monotonous and lacking in necessary visual variation and interest, leading to aesthetic fatigue and psychological burnout [55]. Notably, pastoral landscapes significantly enhance positive emotions even at very low levels of presence. This reflects the unique advantages of natural-agricultural mixed patterns in providing visual buffering and cultural associations [60]. This may also be closely related to the local landscape features of the Wuyi Mountain region, which is characterized by its tea culture.

The influence of artificial elements on emotions exhibits a more complex dual nature, with their positive and negative effects highly dependent on scale adaptability and landscape organization. Among these, the multi-peak response of roads reveals multiple optimal modes of road-landscape integration. A medium road proportion works best because it strikes a balance between the road’s functional role and its landscape presence, ensuring they do not interfere with each other. Narrow or excessively wide roads, leading to significant negative emotions, point to the imbalance of functional safety anxiety and the disruption of natural landscape experience. The multi-peak threshold characteristics of spatial division reflect the differential impact of various division situations on emotional arousal. Minimal spatial division disturbances tend to cause monotony and fatigue, moderate division (such as scattered utility poles) may disrupt spatial continuity and lead to irritability, while high division (such as wall obstruction) exacerbates feelings of oppression. This complexity is particularly evident in linear spaces, where the continuous movement process can amplify the cumulative effect of spatial division disruptions, significantly increasing cognitive load.

Macro-environmental factors collectively form the spatial background for emotional potential. Mid-to-low elevations, gentle to moderate slopes, and south-facing slopes typically create a more optimal balance between microclimate conditions, physical exertion, and scenic comfort, thereby facilitating the generation of positive emotions [61]. Different types of land use and land cover conditions also elicit varying emotional responses [62]. The clustering of emotional hotspots in semi-natural landscapes, such as rice fields and forested areas, highlights the unique value of cultural landscapes that blend ecological aesthetics and human traces in evoking emotional resonance [63].

Overall, emotional experiences along the scenic road are not directly determined by a single landscape element, but are the result of the combined effects of multi-scale, multi-type factors within specific threshold ranges. The existence of nonlinear threshold characteristics reveals that the key to emotional regulation in linear recreational spaces lies not in the simple accumulation of elements, but in the proportions, rhythms, and spatial organization of different elements within the sequence of movement.

### 4.4. Implications for scenic road planning and future research

The nonlinear threshold relationships identified in this study provide several practical implications for the planning and management of the No. 1 Scenic Road. Three aspects deserve particular attention:

(1) Preserve moderate visual openness along key scenic segments. The visual field is the dominant driver of emotional arousal, with emotional responses peaking when openness reaches moderate levels. Planners should preserve strategic view corridors by controlling vegetation overgrowth and avoiding the placement of large structures that block key vistas.
(2) Reduce the visual dominance of artificial elements. Several artificial elements showed unfavorable emotional responses when their visual proportions became excessive, particularly when road-related or spatial-division elements exceeded moderate levels. In core scenic segments, new infrastructure should be designed to minimize visual intrusion by using local materials, low-contrast colors, and compact footprints.
(3) Enhance selective visibility of natural landscape features. Water bodies and pastoral landscapes generate strong positive emotional responses even at low visual proportions. For example, water visibility within the 1% to 2.5% range and pastoral landscape proportions above 2% both yield significant positive effects. A cost-effective strategy for enhancing visitor emotional experience is to create selective visibility of these elements through careful viewpoint design and vegetation management, rather than pursuing large-scale landscape interventions.

While these planning recommendations provide practical guidance for scenic road management, the present findings should be interpreted in light of the experimental setting. The VR-based experiment provided a controlled and immersive environment for evaluating emotional responses, but it could not fully replicate real-world driving experiences. In actual travel, emotional responses may also be influenced by vehicle motion, physical fatigue, soundscape, weather conditions, and other multisensory stimuli that were beyond the scope of this study. Future research could integrate immersive driving simulators, physiological measurements, and field-based experiments to further validate and extend the applicability of the proposed planning strategies under real-world conditions.

## 5. Conclusion

This study shows that emotional experiences along scenic roads are not static point or area phenomena as traditional emotional mapping, but rather a spatial distribution that unfolds along the linear path. Emotional fluctuations along the scenic road exhibit significant scale dependence and spatial hierarchical characteristics. At the main road scale, emotional responses are highly path-dependent, forming a band-like clustering structure centered around the road within a close range, while at larger spatial scales, the distribution gradually transforms into a discrete pattern that resembles an area-based distribution. Meanwhile, positive and negative emotions are not simply oppositional in certain core sections but are simultaneously activated in a coexistence form, reflecting the complexity and multi-directionality of emotional experiences in linear recreational spaces. This finding breaks the simplified assumption of understanding spatial experiences based on a single emotional orientation and provides a new framework for understanding the spatial organization of emotions in linear public recreational spaces.

On this basis, the study further reveals the nonlinear driving mechanisms behind emotional spatial distribution. Emotional responses are not linearly determined by a single landscape element, but are the result of the combined effects of natural elements, artificial elements, visual elements, and macro-environmental factors within specific threshold ranges, with the proportions of these elements and spatial organizational structure playing a key role in emotional arousal. These insights provide scientific support for the human-centered planning and refined management of national park scenic roads, and lay the theoretical and practical foundation for extending the emotional mapping method from localized areas to long-distance linear spaces. Future research should further combine multi-sensory emotional perception and real-time behavioral tracking techniques to validate and expand the linear emotional spatial structure under different populations and contexts.

## Supporting information

S1 Table Predictive performance of the preliminary GLMM on the training and test datasets. To evaluate the suitability of different prediction approaches, a preliminary Generalized Linear Mixed Model (GLMM) was constructed for comparison with the MaxEnt model. The following supplementary materials summarize the predictive performance and diagnostic results of the GLMM, which served as a reference for model selection.
(PDF)

S1 Fig Diagnostic plots of the preliminary GLMM used for model comparison. To evaluate the suitability of different prediction approaches, a preliminary Generalized Linear Mixed Model (GLMM) was constructed for comparison with the MaxEnt model. The following supplementary materials summarize the predictive performance and diagnostic results of the GLMM, which served as a reference for model selection.
(TIF)
