# Supplementary material for: Mapping emotional patterns in linear recreational landscapes: Spatial structure, positive-negative co-occurrence patterns, and nonlinear drivers
Source: PLoS One. 2026 Sep 8;21(9):e0356187. doi: 10.1371/journal.pone.0356187 (PMC13552935; doi:10.1371/journal.pone.0356187)
Supplement: Supplementary file 1 [file pone.0356187.s001.pdf]

S1 Table. Predictive performance of the preliminary GLMM on the training and test datasets.

**Group**	**R^2**	**MSE**	**RMSE**	**MAE**
Training set	0.9113	0.0028	0.0536	0.0429
Test set	0.4597	0.0117	0.1086	0.0855
